# Genomic assemblies of newly sequenced *Trypanosoma cruzi* strains reveal new genomic expansion and greater complexity

**DOI:** 10.1038/s41598-018-32877-2

**Published:** 2018-10-02

**Authors:** Francisco Callejas-Hernández, Alberto Rastrojo, Cristina Poveda, Núria Gironès, Manuel Fresno

**Affiliations:** 1 Centro de Biología Molecular Severo Ochoa, Consejo Superior de Investigaciones Científicas, Universidad Autónoma de Madrid, Cantoblanco, Madrid, Spain; 2 Instituto Sanitario de Investigación Princesa, Madrid, Spain

## Abstract

Chagas disease is a complex illness caused by the protozoan *Trypanosoma cruzi* displaying highly diverse clinical outcomes. In this sense, the genome sequence elucidation and comparison between strains may lead to disease understanding. Here, two new *T. cruzi* strains, have been sequenced, Y using Illumina and Bug2148 using PacBio, assembled, analyzed and compared with the *T. cruzi* annotated genomes available to date. The assembly stats from the new sequences show effective improvement of *T. cruzi* genome over the actual ones. Such as, the largest contig assembled (1.3 Mb in Bug2148) in *de novo* attempts and the highest mean assembly coverage (71X for Y). Our analysis reveals a new genomic expansion and greater complexity for those multi-copy gene families related to infection process and disease development, such as Trans-sialidases, Mucins and Mucin Associated Surface Proteins, among others. On one side, we demonstrate that multi-copy gene families are located near telomeric regions of the “chromosome-like” 1.3 Mb contig assembled of Bug2148, where they likely suffer high evolutive pressure. On the other hand, we identified several strain-specific single copy genes that might help to understand the differences in infectivity and physiology among strains. In summary, our results indicate that *T. cruzi* has a complex genomic architecture that may have promoted its evolution.

## Introduction

*Trypanosoma cruzi* is a highly polymorphic parasite that belongs to the order Kinetoplastidae, being the causative agent of Chagas disease or American Trypanosomiasis, a chronic illness and one of the most neglected tropical diseases^1,2^. Chagas disease is endemic in Latin America but due to migration of infected people, this disease has been extended to non-endemic areas, such as the European Union and the United States, making of Chagas disease a serious public health problem^3–5^. Currently, 7 to 10 million people are thought to be infected and 10,000 to 14,000 deaths per year are caused by this disease^2^.

Chagas disease occurs in two phases: acute and chronic, that can be asymptomatic for decades and evolve towards the most aggressive symptomatic phases characterized by cardiac and/or digestive clinical forms^1,2^. This broad spectrum of clinical manifestations had been related to parasite and host genetic variability^6,7^. Thus, there is a significant variation in the size, structure and ploidy among the different parasite strains, although *T. cruzi* is usually described as diploid^8–13^. Genes in trypanosomatids do not present promoter regions to regulate gene expression. Contrarily, gene duplication and mRNA stability, among others, are likely the possible mechanisms to control gene expression^14^, as it has been well documented in other Kinetoplastidae as *Leishmania ssp*. and *T. brucei*. This phenomenon explains the selection of highly repetitive (or low complexity) genomic content and variations through evolution^9,15–18^, likely originated by the accumulation of transposable elements, high prevalence of tandemly repetitive sequences (involved in gene divergence) and repetitive short sequences in chromatin remodeling^19,20^. Thus, the relationship between short repeats, tandem repeats and genome size has been of interest because some repeated sequences are significantly expanded in length during evolution^20–22^. In addition, the genomic repetitiveness in other species has been associated with natural selection, where low complexity plays a critical role in the control and/or mediation of gene expression and variability^23,24^. In the 19^th^ century, Haldane and Stadler suggested that gene duplication and divergence might be favorable because of the possibility to produce genes without any disadvantage to the organism, in the sense that multi-copy genes would be less susceptible to damaging mutations demonstrating that polyploid unicellular organisms were less susceptible than their diploid congeners to irradiation^25^.

In 2009 a classification based on the genetic structure (including genomic and mitochondrial kinetoplastid DNA) was proposed that describes the existence of six separated clusters or discrete typing units (DTUs) of *T. cruzi* isolates, or strains, named from TcI to TcVI, where TcV and TcVI have a hybrid evolutionary origin, with TcII and TcIII as putative parents^26^. However, this classification has been questioned after a systematic phylogenetic tree reconstruction based on most frequent mitochondrial *T. cruzi* genes in genome databases, which showed the existence of three significant clusters named as mtTcI, mtTcII and mtTcIII instead 6 DTUs (7 including B7 belonging to TcBat) at least with the actual data and phenotyping techniques^27^.

An extensive number of publications about this disease suggest significant phenotypic variation and different behavior both *in vitro* and *in vivo* in terms of pathophysiology, virulence, tropism and immunological responses, which strongly difficult the development of vaccines or new drugs against this disease for which available treatments have limited efficacy and side effects^2,28–30^.

Despite these limitations, the publication of several *T. cruzi* genomes has represented an important advance for the understanding of the complexity of this parasite^31^. To date, there are available public genomes from Dm28c, JRCl4 and Sylvio X10 (TcI), Esmeraldo (TcII), Tula, CL Brener Esmeraldo-like “BEL” and non-Esmeraldo-like “BNEL” (TcVI), and B7 (belonging to *T.c marinkellei*) strains, but not from TcIII, TcIV and TcV strains. However, whole genomes or whole chromosomes (Mb of length) cannot be read at a glance with any actual technology, thus millions of fragmented sequences must be assembled in longer fragments named contigs, and when possible, in scaffolds as result of the in tandem union of contigs. Besides, some important questions have raised from genome organization that are still unresolved, such as: (1) the reason and consequences of different genome sizes between strains, (2) karyotype polymorphism even between strains belonging to the same DTU, (3) the presence of tandem repeats and low complexity regions along the whole genome and (4) its relationship with the physio-pathogenesis of the disease. In this study, two *T. cruzi* strains have been sequenced by Next Generation Sequencing (NGS) technologies, assembled with new software programs, analyzed and compared to available genomes. The TcII Y strain was chosen due to their importance in terms of virulence, infectivity and disease development in experimental models^32–34^, while Bug2148 belonging to TcV, was chosen because it is more frequently associated to vertical transmission^35^.

## Results and Discussion

### Genome assembly and performance

After quality and length trimming process of the sequences, about 18 million of paired reads for the Y strain were obtained (mean read length: 240 pb), and 757,037 reads for Bug2148 (mean read length: 14 kb), which correspond in both cases to ~10 GB of information and to more than 100x read depth coverage (RDC) for the predicted haploid genomes^36,37^. Contigs from SPAdes assembler for the Y strain (from Illumina) with low coverage (<10X) were removed from the assembly, to reduce chimeric sequences and pseudogenes resulting in 10,127 total assembled contigs (Table 1). In the case of Bug2148 (from PacBio), a total of 934 assembled contigs were obtained with a QV (Quality Value scores) around 99.99% (assembly accuracy) after HGAP’s assembly pipeline (Table 1). Genome for the Bug2148 cl1 strain was assembled in a low number of total contigs, lower than almost all available *T cruzi* strains to date. PacBio technology has the capacity of sequence longer reads (until 20 Kb) which in the case of *T. cruzi* genome project may represent a great advantage avoiding complex and/or repetitive regions and facilitating the final assembly. Both new assembled genomes contain around 50% of GC in agreement with previously sequenced *T. cruzi* strains. Based on the total assembled bases, the predicted haploid genome and the total contigs, Bug2148 is probably the most complete haploid *T. cruzi* genome sequenced to date (Table 1).

**Table 1:** Summary of *T. cruzi* assembled genomes.

Strain	DTU	Size (MB)	GC (%)	Contig N50 *	Max lenght (PB)	Min lenght (BP)	Mean contigs (BP)	Contigs	Scaff level*	Coverage (%)	Sequencing method
Dm28c	I	27.34	53.33	78,389	462,134	2,004	22,601	1,210	Scaff*	63	454*
Jrcl4	I	41.48	52.45	83,591	828,981	200	2,709	15,312	Scaff	69	454
Sylvio X10	I	38.58	52.91	2,307	72,500	202	1,428	27,019	Contigs*	30	454 + illumina
Y	II	39.34	51.43	11,782	304,87	500	3,885	10,127	Contigs	71	Illumina
Esmeraldo	II	38.08	52.09	66,229	483,664	200	2,409	15,803	Scaff	60	454
Bug2148	V	55.22	51.63	196,760	1,305,792	585	59,129	934	Contigs	68	PacBio*
Tula	VI	83.51	50.63	7,772	242,476	200	1,826	45,711	Scaff	50	454
BNEL	VI	32.52	43.94	870,934	2,371,736	77,958	793,392	41	Chrom*	14	454 + BAC*
BEL	VI	32.52	40.35	870,934	2,371,736	77,958	793,391	41	Chrom	14	454 + BAC
B7	VI	38.65	52.87	25,781	335,615	235	2,302	16,783	Contigs	30	454 + illumina

*Contig N50: is a statistic median such that the 50% of the entire assembly is contained in contigs equal to or larger than this value. *Scaff level: Scaffolded level assembly. *Chrom: Chromosome level assembly. *454: Roche 454. *PacBio: Pacific Biosciences. *WGS: Whole Genome Sequecing. *BAC: Bacterial Artificial Chromosome. Previous *T. cruzi* genomes available at https://www.ncbi.nlm.nih.gov/genome/genomes/25? Newly sequenced genomes are underlined.

As an indirect measure of the general genome complexity that may explain the miss-assembly level for the new genomes, the %GC was calculated for each assembled contig (S1 File). In both cases and as it was expected, longest contigs contain around 50% GC composition meanwhile shorter ones possess more variable distribution, indicating assembly breakages on low complexity (more repetitive) genomic areas in agreement with typical *de novo* assemblies (Fig. S1). This pattern of GC distribution is dramatically different for the two new assembled genomes due mainly to the inherent technology differences.

The mean RDC obtained for Y strain assembled genome (71X) is the best mean coverage among all strains actually sequenced and a little higher than for Bug2148 (68X). Unfortunately, complete chromosome reconstruction from short reads produced by Illumina (250 pb for this experiment) is not possible, resulting in very fragmented genomes even in the order of thousands of pieces (Table 1). This is a big problem in complex genomes like Trypanosomatids, and leads to over-, under- or mis-representation of genes or complete chromosomic locations.

In this regard, Arner *et al*.^24^ suggested that in *T. cruzi*, the copy number of some conserved genes can be used as misassembly control. For example, monoglyceride lipase gene was predicted to have around 50 copies in several different strains from different DTUs, but despite that, it has been annotated only once for CL Brener and Sylvio X10 genomes (and not found on remaining annotated strains). However, we found 4 copies in the Y strain and 30 in the Bug2148 sequence, which may also confirm the high level of assembly performance in our analysis (Fig. S2).

### Gene prediction and functional analysis

Contigs from both new assemblies shorter than 500 pb were removed from the total assembly in addition to RDC filter (10X). Next step was predicting genes across these filtered sequences and predict their theoretical function. A total of 33,306 and 20,058 complete ORFs were obtained for Bug2148 and Y, respectively, being the last figure similar to previous *T. cruzi* gene predictions (belonging to DTUs I and II). However, the number of ORF in Bug2148 was much higher.

Functional annotation of predicted ORFs was performed following two different approaches, first, gene families were defined by self-Blastp and MCL clustering algorithm and second, function was defined by the best reciprocal BLAST against the complete database of protozoan annotated proteins. The minimum e-value was set to 1e-5 and identity ≥50%, genes without blast hit were annotated as “Hypothetical protein”. After gene clustering process, we obtained a total of 10,549 and 10,674 clusters for Bug2148 and Y, respectively, which correspond to the total and theoretical genes families. For about ~50% of the total predicted genes it was not possible to find a theoretical function, as it is expected for Trypanosomatids (Table 2).

**Table 2:** Percentage of hypothetical protein content across *T. cruzi* annotated strains.

Strain	Hypothetical Protein (%)
Dm28c	64.26
Sylvio X10	49.50
Y	56.94
Bug2148	53.25
BEL	51.53
BNEL	51.55
B7	50.60

**Figure 1:**
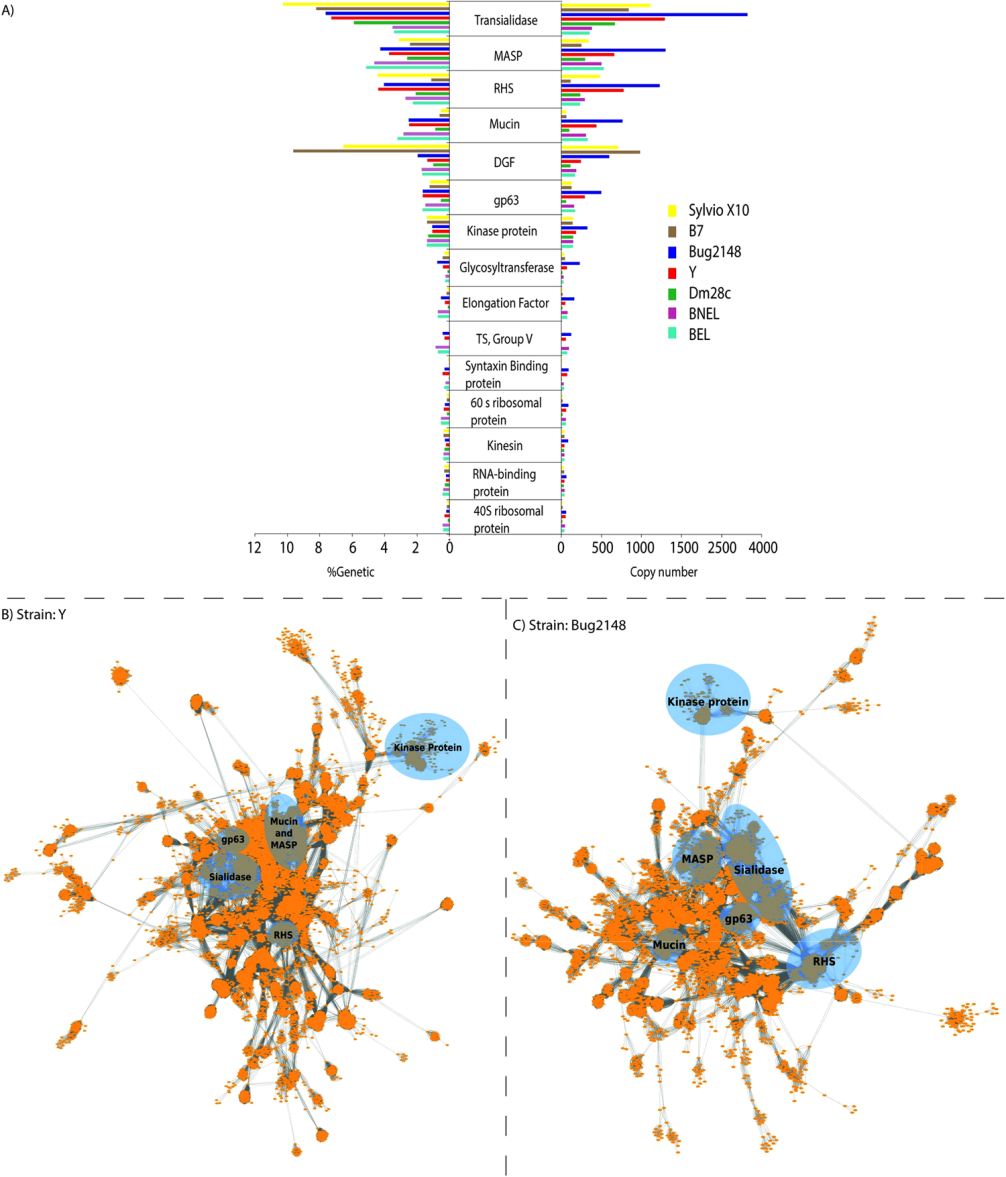
Protein families in *T. cruzi*. (**A**) On the left, the genetic percent that represents the gene copy number found (for the top 15) based on the total annotated genes of Y and Bug2148 and public available genomes. On the right, the copy number for these genes along the entire genome. (**B**) Gene families clustering for the Y strain including hypothetical proteins. (**C**) Gene families clustering for the Bug2148 strain including hypothetical proteins. Most abundant families are remarked by blue circles.

Next step was to perform a genetic diversity analysis for all *T. cruzi* genomes actually sequenced and annotated (available without restrictions at Tritryp data base in 10/06/2017: TcI (Sylvio X10, Dm28c), TcII (*new Y), TcV (*new Bug2148 cl1), TcVI (CL Brener BEL and BNEL) and B7. For the new assembled strains ORFs with similarity to known functions were collapsed into families, and protein families for the remaining strains were extracted from their annotated proteins. Results for the 15 more abundant families, which also constitute about 25% of the total genetic content including hypothetical proteins (Y and Bug2148 24.38% and 26.06%, respectively), are shown in Fig. 1A.

In other words, from the total predicted ORFs (33,306 and 20,058 for Bug2148 and Y, respectively) we have identified the total gene families or gene types (10,549 and 10,674 clusters for Bug2148 and Y, respectively) based on its theoretical function. About 50% of the total gene families correspond to hypothetical proteins (also hypothetical conserved) as it was expected. For the remaining 50% of the total genes we have identified 1,589 different functions where the 15 more abundant represent the 25% (approximately) of the total assembled genome (complete data in S2 File), which is lower than previous suggestions of 50%.

Gene clustering for Y strain (Fig. 1B) and Bug2148 (Fig. 1C) highlights the differences between the two new genomes. We confirmed *in silico* the level of divergence between the most abundant protein families that, in some cases have caused its sub-classification. There was a certain degree of divergence between strains respect to the top 5 most abundant known family clusters (in blue circles). This will lead to a better understanding of its relationship with disease development and/or the complex parasite life cycle. Interestingly, we have found that hypothetical proteins may constitute an important and unknown genetic structure of *T. cruzi* since these proteins correspond to at least the 50% of the total *T. cruzi* coding genome that grouped in many clusters and they may constitute new functionally important new families of up 700 copies.

The most abundant gene families in *T. cruzi* are these coding surface protein families such as: Trans-sialidase-like (TS), Mucin, Mucin Associated Surface Protein (MASP), gp63, others related to genetic expression like retrotransposon hot spot (RHS) and the Dispersed Gene Family proteins (DGF) which function remains not well understood. The comparison between available sequences both in absolute copy number as well as percentage of that assembled genome revealed interesting differences. The predicted copy number of genes within TS-like family was around 1,400 for Y as it was expected (1,419), and almost the same for Sylvio X10 (1,321), in agreement to previous estimations^38^. In contrast, a higher copy number (2,325) was found in Bug2148 (Fig. 1A, right). However, the percentage that this family represents for each strain from the total genome (coding sequence) is quite different (over to 2% of the total genome, Fig. 1A, left). Some reasons may explain those differences. Although Bug2148 has the highest TS copy number compared to all available strains, the percentage of TS genes respect to the total is very similar to Y and B7 (about the 8%). Thus, differences in copy number are likely due to hybrid origin of Bug2148. As it has been suggested before, the TS family could be under or overrepresented due to assembly and technology limitations, including the Y strain, but also, the same tendency may also apply to Mucin, MASP and RHS families. Some genes families encoding the most abundant proteins in *T. cruzi* have been found near repetitive telomeric locations, which may cause collapsed assemblies and therefore very fragmented genomes (shown in Illumina assemblies, Fig. S3)^39^. Moreover, other variables related to the complex kinetoplastid genome such as remarkable karyotype plasticity and aneuploidy are playing decisive roles in sequencing projects^9,28,40^.

MASP proteins contain N- and C-terminal domains that encode a putative signal peptide and a GPI-anchor addition site^41^. They constitute about 6% of the parasite haploid genome and are involved in immune evasion^42^. We found here that this figure correlates with the amount predicted for Dm28c (3.5%), TcVI from CL Brener (3.5%), and B7 (3.0%) and the percentage was similar in our Y (3.7%) and Bug2148 (3.9%) strain sequences.

However, for some other multigene families the situation is rather different between strains (Fig. 1A). For example, genes encoding retrotransposon hotspot (RHS) activity, a protein associated to telomeric locations and replicative processes, are as abundant as MASP in Bug2148, Y and Sylvio X10 but much lower in B7, which clearly is not infective in humans. In addition, our analysis showed that mucins are the fourth largest *T. cruzi* gene family (all strains considered), and not the second as it was previously supposed based only on the genetic profile evidence from the CL Brener haplotypes, which were the first annotated genomes. This family is much less represented (2–3 fold) in available Sylvio X10 and Dm28c genomes than in Bug2148, Y and CL Brener. Opposing results were obtained for dispersed gene family proteins (DGF), proteins believed to act similarly to integrins, being the largest family in B7 and also highly abundant in Sylvio X10, 3–4 fold more in percentage than the rest. This family was considered the 5^th^ largest family up to date in *T. cruzi*^42^, however, our results showed it may be the largest in the nonpathogenic B7 and the second for Sylvio X10 where constitutes about the 7% of its total coding genome. In addition, this family was suggested to be genetically sub-classified into at least three groups due to its susceptibility to gene recombination promoting gene divergence^43^. However, *in silico* insights showed differential degrees of gene diversity between strains (Fig. S4), more than a general classification clade suitable for all strains. Syntaxin-binding protein family, involved in the regulation of synaptic vesicles docking and fusion processes, is also differentially represented. According to our results, Sylvio X10, Dm28c and B7 have very low percentages of this family, compared with the rest of strains and mainly found in our newly assembled genomes. The reasons and implications of this phenomenon are unknown.

Thus, our results and analysis suggest that to date, the content and diversity of the six more extensive gene families (TSs, MASP, RHS, Mucins, DGF and gp63) is related to a strain-specific genetic profile, the accuracy of its assembled genome, and genomic plasticity variable among strains more than to a general genomic structure pattern. Since these families play multiple roles in virulence, evading vector’s and host’s defensive mechanisms and are involved in parasite invasion of host cells, the relationship between diversity and expression with strain biological features deserve further and deeper studies.

### Trans-sialidase activity: subfamilies

In *T. cruzi*, TS genes comprises a large family of over 1,400 genes sharing the VTVxNVxLYNR motif and TS family members are localized on the membrane surface of metacyclic, blood stream trypomastigotes and intracellular amastigote being the principal gene family involved with host parasite interaction processes^44–46^. Some TS catalyze the transference of sialic acid molecules from host glycoconjugates to acceptor molecules placed on the parasite surface^47^, and therefore has been thought to play crucial roles in parasite survival and the establishment of effective infections. In addition, TS is the most polymorphic and complex surface protein family, and also the most abundant genetic family (expressed in the surface) in *T. cruzi*. Genes encoding TS or TS-like genes are actually classified in 8 groups^46^, where these groups are defined by specific motifs and show specific activities. Moreover, critical residues necessary for catalytic activity have been identified just in a few genes. Despite this sub-classification and the importance of the family members as virulence factors, most of these subfamilies were not identified in all *T. cruzi* genomes available to date.

**Figure 2:**
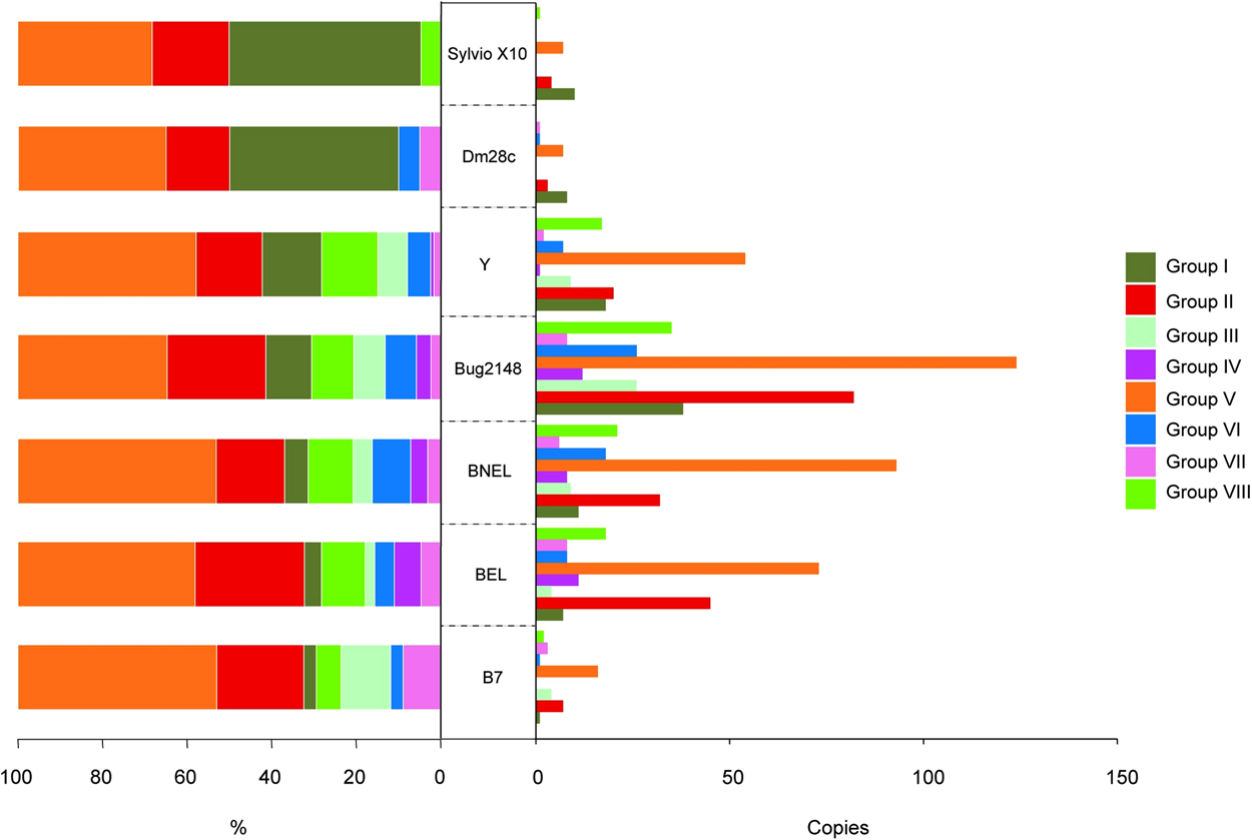
TSs sub-families’ distribution (groups I to VIII) in the newly assembled and public genomes. On the left, percent of each TS group based on the total genes that constitutes the complete TS family for each strain. On the right, number of copies for TS groups by strain.

Therefore, we analyzed the content of TS subfamilies in all available genomes. Genes from TS group V containing Asp-Box motif (SxDxGxTW) are the most expanded group, both in absolute copy number and % of total TS-like genes, for almost all the strains, except for Dmc28c and Sylvio X10 (of note they belong to the same DTU, TcI) (Fig. 2). This TS group has been associated to antigenic variation, which allows the parasites to adapt to the host environment^46^. TS group II, which does not have enzymatic activity but contains gp85, gp82, gp90 members and other glycoproteins mainly implicated in host cell attachment and invasion, is the second more expanded cluster for Bug2148, BNEL and BEL (124, 93 and 73 copies, respectively).

Interestingly, in the 2 TcI strains Dm28c, Sylvio X10, and in B7 genomes, we could not find TS group IV, although this cannot absolutely discard that they do not exist. It is very important to note that TcI strains including Sylvio X10 are the closest relative to B7 corroborating the high evolutionary relationship of TcBAT with TcI and the emergence of TcBAT genotype in the natural history of *T. cruzi* taxon^48^. TS group V in Y strain showed a low frequency compared to BEL, BNEL and Bug2148, that have the highest percentages and copies.

On the other hand, we found that TS group I, which contains the enzymatically active sialidases TCNA (*T. cruzi* neuraminidase) and SAPA (Shed acute-phase antigen) is not as abundant as predicted, since it was thought to be present in 60–80 copies per haploid genome^49^, but according to our analysis of the CL-Brener genome this family is clearly much lower (BNEL 11 copies, BEL 7 copies). In the case of the remaining strains we found that is well represented in the Bug2148 (38 copies), and lower copy number, 18 in the Y strain, 8 and 10 copies for Dm28c and Sylvio X10, respectively. Thus, TS Group I is found in all strains but there are clear differences in copy number and consequently in percentage.

TS Group III includes mostly regulatory proteins that inhibit the alternative and classical complement pathways being FL-160 the best representative^50^. Interestingly, we did not find members of this family in Sylvio X10 and Dmc28c (again the 2 TcI strain). Since FL-160, complement regulatory protein (CRP) family has been also involved in complement system evasion, this may indicate that some strains are more prone than others to evade complement lysis^51^. Therefore, the present gene elucidation and enrichment for this relevant family may contribute to a better understanding of its function.

TS Group IV, which function remains unknown (Tc13 is the representative sequence), was found only present in the CL-Brener genome and in the new Bug2148 strain, being practically absent on other strains that have their genome sequence were more fragmented. Finally, the TS group VII represented by the TS family motif (xTVxxVxLYNx) was found as one of the smallest group across the annotations. These results confirm that this group is actually not abundant in agreement with previous analysis^9^ including the CL-Brener and Bug2148 genomes.

### Chromosomal structure in *T. cruzi*

*T. cruzi* has a highly plastic genome, an unusual gene organization and complex mechanisms for gene expression such as polycistronic transcription, RNA editing and trans-splicing^16,52^. Furthermore, due to the lack of mechanisms controlling transcription initiation, subsets of genes must be post-transcriptionally co-regulated in response to extracellular signals. Nowadays, the organization of those genetic subsets remains undefined. More than 50% of the *T. cruzi* genome consists of tandemly repeated sequences and even in diploidy, the parasite has a high variation in chromosome number and even aneuploidy arrangements^9,12,24^. These genome size variations could be related to gene copy number (including pseudogenes and/or non-coding regions), and different reasons had been suggested for this phenomena such as, evasion of the host immune response, the absence of transcription control mechanisms and the complex biological needs through its life cycle in the vector and in the host^53,54^.

**Figure 3:**
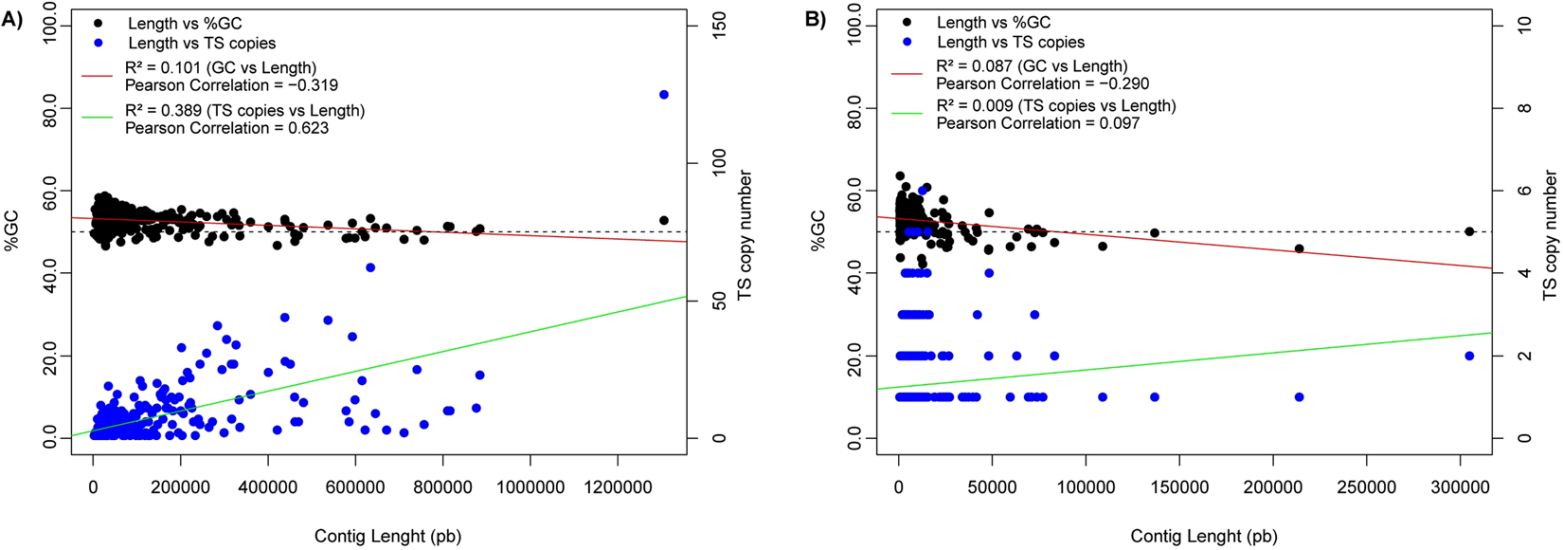
TS-cc length and %GC distribution of the new assembled genomes. Plots of the TS copy number *versus* contig length (blue circles), TS copy number versus %GC (black circles) and Pearson correlations between TS copy numbers and contig length (green line) and between TS copy number and %GC (red dotted line) for Bug2148 (**A**) and Y (**B**) strains.

It has been proposed that some of the most expanded gene families in *T. cruzi* are localized at telomeric and subtelomeric regions that are subject to continuous evolutionary processes. These sites may act as a site for DNA recombination, expansion and generation of new gene variants; this may include DGF, RHS, TS and other acetyltransferases. In particular, TS-like genes have been mainly located near telomeric regions, promoting the generation of new gene variants via non-homologous recombination, as a mechanism by which the parasite evades the host immune response^39^. However, this important evolutionary advantage for the parasite may be causative of *in silico* miss- or over-representation of this complex family through the assembled genomes available to date. This could be due to collapsed assemblies in complex regions, which in some cases correspond to telomeric sequences with specific tandem repeats or other short repetitive genomic motifs. In this sense, we have analyzed the %GC in TS-containing contigs (TS-cc) including pseudogenes, as indirect measure of complexity. Results showed that in both new genomes, short TS-cc have a slightly higher GC content, which negatively correlated to their length (pearson correlation of −0.319 and −0.29 for Bug2148 and Y, respectively) (Fig. 3). This pattern confirms that assemblies have collapsed because of highly repetitive sequences mainly GC-enriched, where telomeric motifs may play an important role. However, *T. cruzi* genes (most of them) are packed in tandem arrays or polycistronic transcription units (PTUs) where ORFs may correspond to different functions^55^ more than copies in tandem for the same gene. Thus, we have also analyzed the correlation between TS copy number and length in TS-cc, demonstrating that it actually exists in Bug2148 genome specially for contigs about 200–700 Kb long which contains up to 70 TS copies (Pearson correlation of 0.623), but for Y strain there was no significant correlation due to assembly fragmentation, where the longest contig (also identified as TS-cc) barely reached 300 kb.

**Figure 4:**
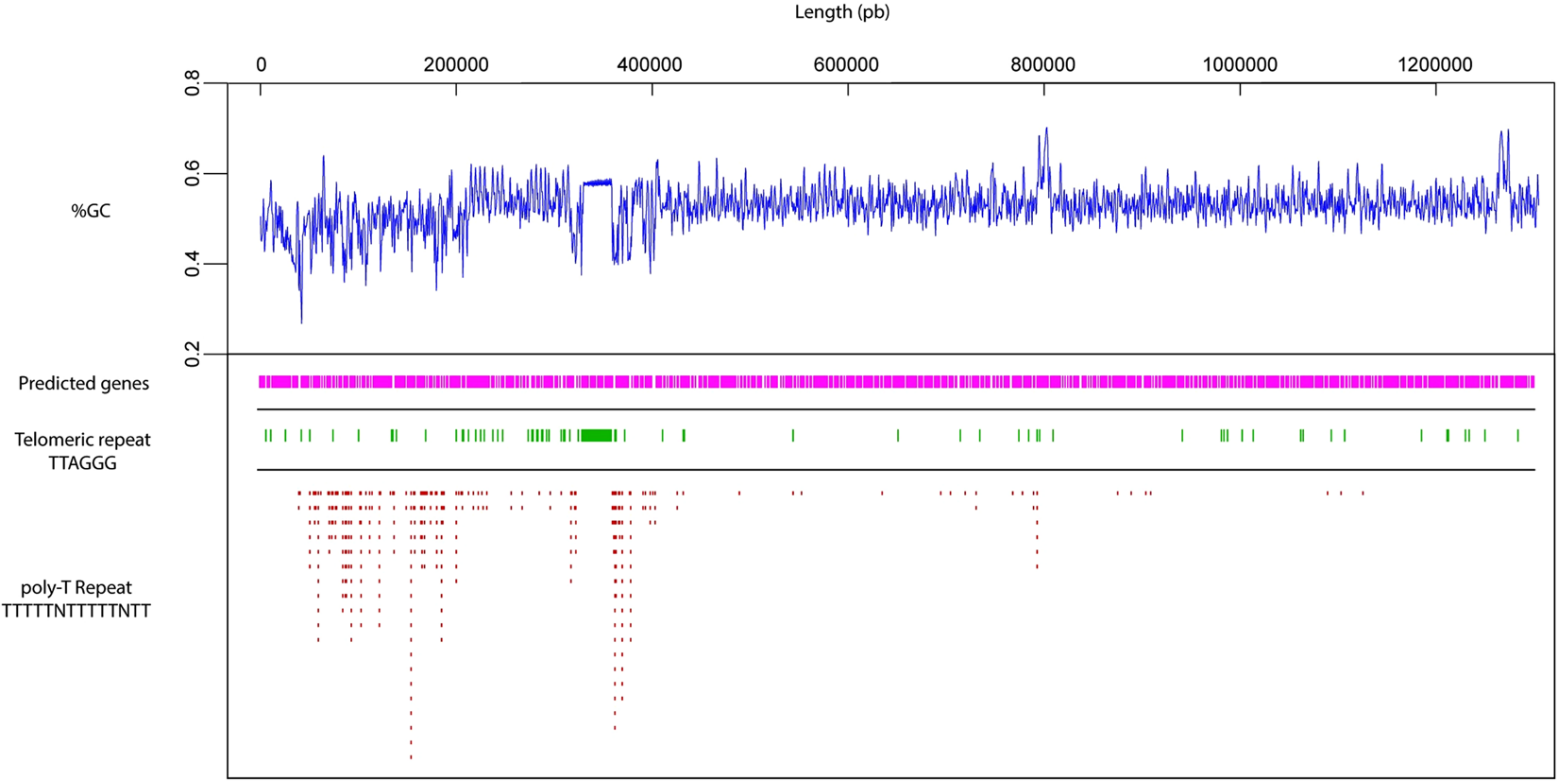
Relation between %GC and telomeric repetitive motifs. %GC variation across largest contig 1.3 Mb (“chromosome-like”) from Bug2148 assembly and its relation with the presence of telomeric and poly-T repetitive motifs. TS gene family location at sub-telomeric regions is indicated.

Once we had strong evidence of TS-cc assembly is affected by complexity, we performed the localization of the specific telomeric repeat (TTAGGG) and poly-T described for *T. cruzi*^56,57^ taking as reference the largest contig in Bug2148 (about 1.3 Mb), which correspond to almost the largest entire predicted chromosome in *T. cruzi* (Fig. 4) and also the most important TS-cc (containing 120 copies). This analysis also demonstrates that variations in %GC, at least across this chromosome-like sequence, are directly related to repetitive motifs such as telomers and poly-Ts, in agreement to previous speculations^57^, but additionally suggesting that telomeric locations may be 400 kb long. Therefore, this locus has not been previously detected in practically any of the available *T. cruzi* genomes to date, likely due to technology limitations since the De Bruijn Graph algorithms from short sequences used are not the best for reconstruction of long complex sequences.

Interestingly, we found tandem repeats of casein kinase protein (CK) inside the longest tandem chromosomic repeat. This protein is common across all eukaryotic cells, belongs to the multipotential Ser/Thr family and possess the ability of phosphorylate a wide variety of cellular proteins, being one of the most important protein kinases in cell function^58^. However, its specific role in this kinetoplastid remains poorly understood and the reason for this special chromosomal location deserves further and deeper studies.

For the first time, we have described a closer insight to genetic composition in *T. cruzi*, and therefore, we can also confirm that the most important protein families (at least the known ones) were underrepresented in previous genomic sequences due to technical sequencing limitations and/or incomplete assemblies.

**Figure 5:**
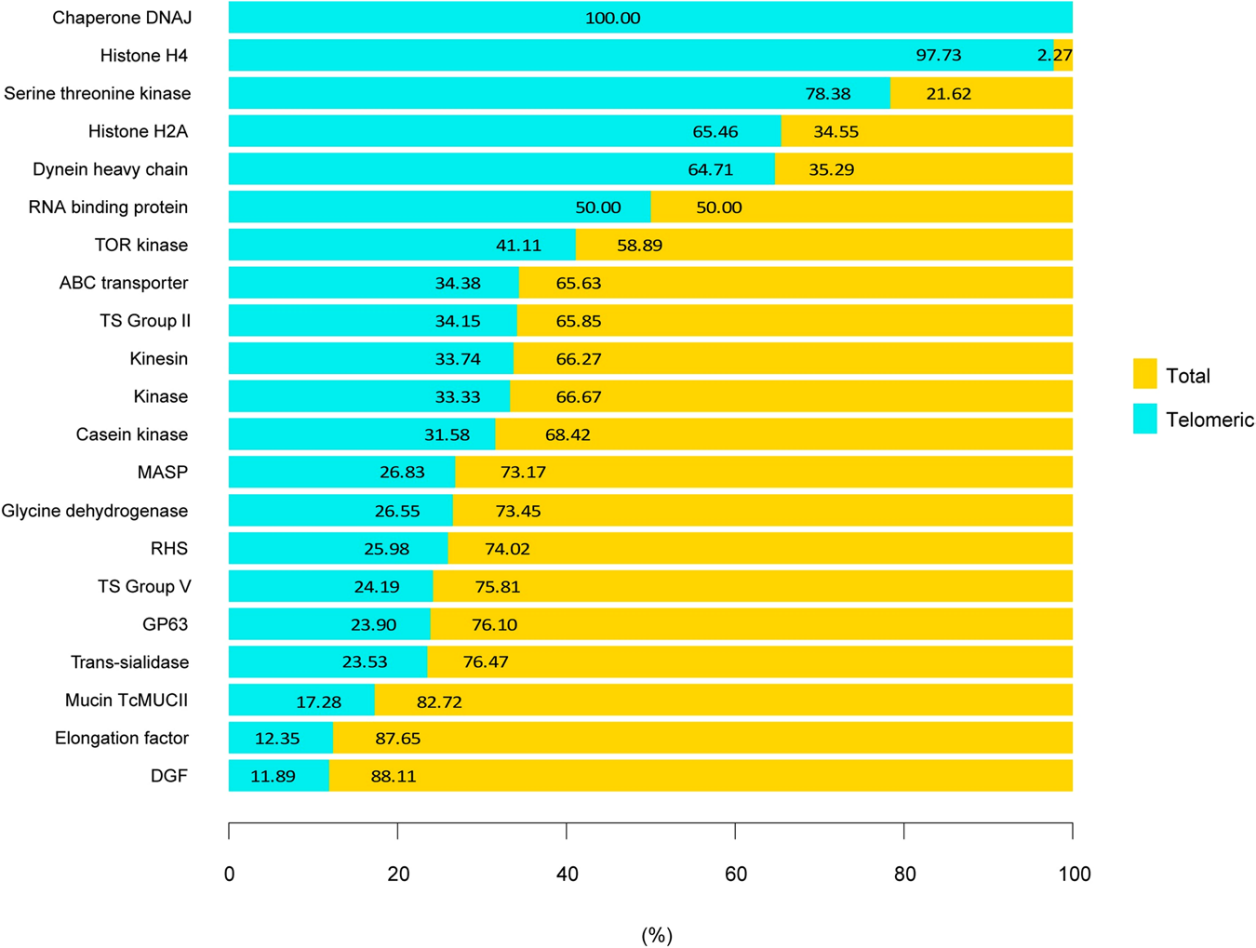
Annotated genes in contigs containing repetitive telomeric motifs. Percentage of annotated genes (by family) near telomeric repeats in Bug2148. Contigs containing telomeric motifs and annotations were not found on Y strain genome.

Additionally, we have analyzed contigs containing 20 or more telomeric repeats in tandem in the complete genomes and extracted their annotated proteins (Fig. 5). Previous analyses had suggested that about the 9% of TS, 12% of DGF-1, and 19% of RHS were located in this chromosome ends^39^. However, our analyses *in silico* suggests that this figures are underestimated and that chromosome ends include some of the most expanded (with known activity) gene families. Moreover, in agreement to previous analysis we found that about 25% of the second bigger family (MASP) are located in hot-spots in internal chromosomal sites which allow different evolutionary mechanisms as telomeric recombination^41^.

Surprisingly, our results show that other protein families, typically considered as less abundant, are located at this complex chromosomic region such as the histones H4, H2A, and the chaperone DNAJ, indicating that these families may be also prone to constant evolution. As it has been described before^59^, *T. cruzi* histones present some particularities, mainly at the sequence level, such as high divergence from canonical histones, that in the majority of eukaryotes are highly conserved. In addition, in *T. cruzi*, each histone (canonical and/or variants) is represented by more than one gene. Those coding proteins are described as isoforms, which also may produce unique post-transcriptional modifications marks that are considered essential for chromatin assembly and/or remodelling required in transcription and replication, and in consequence, suggest the existence of essential epigenetic mechanisms in this kinetoplastid^59^ that may be also in constant evolution. On the other hand, the complete telomeric chromosomal localization of chaperone DNAJ may explain the low sequence similarity between its gene copies, which is calculated to be around 15–60%.

As we have mentioned before, about 50% of the *T. cruzi* genome is composed of a considerable variety of repeats or complex sequences such as divergent and multi-copy gene families. However, other types of complex non-coding sequences can be found across chromosomes (i.e. genes, SIRE, VIPER, LTR, RLE, SER, etc.), which may also critically affect sequencing projects. Thus, the level of miss-assembly for all those *T. cruzi* genes located within these repeats in the complete genomes available to date is still considerable. In this work, Y strain genome that was sequenced and assembled from short reads (250pb) could have been affected by these obstacles and as a consequence we just found 11 contigs with more than 20 telomeric repeats, where the most abundant proteins located around this motifs (excluding hypothetical proteins) were 3 kinases and one CDS encoding TS activity.

**Figure 6:**
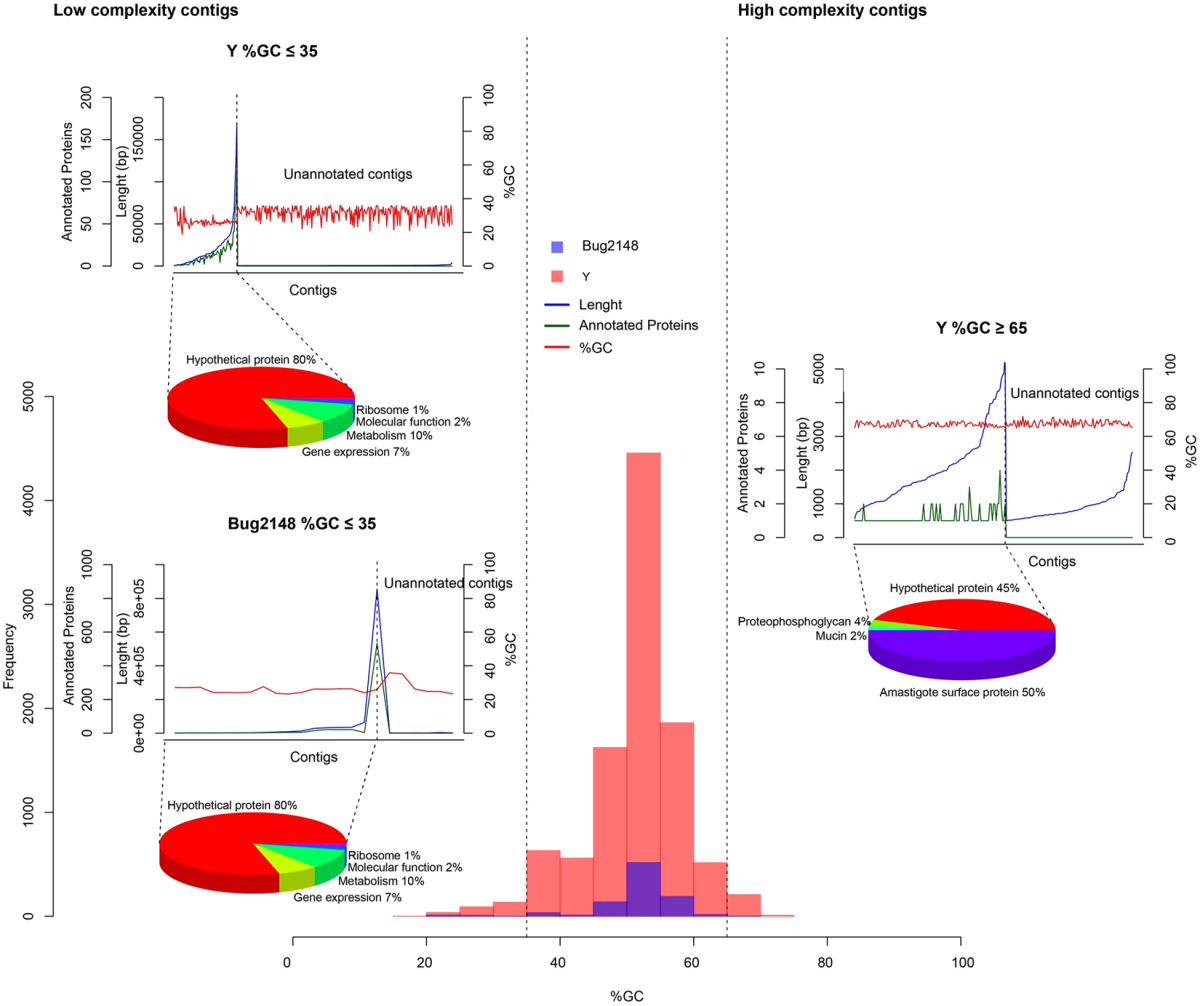
%GC distribution for Y and Bug2148 genomes and genetic composition in the low and high complexity locations. Histogram shows the %GC distribution by contig assembled for the two new genomes, extra graphs shows the genetic annotations on low (lower than 35%) and high (higher than 65%) complexity contigs. (Complete information: File S3).

On the other hand, %GC content in some species is also correlated with a number of genomic features potentially relevant functionally such as: gene distribution, transposable elements, methylation rate, and expression levels. GC-rich genes are more efficiently expressed. Genes located in GC-rich regions tend to be also GC-rich in their coding sequences. Also, the average of GC content is higher at silent sites than in neighboring non-coding region, suggesting that high %GC in coding regions could confer some selective advantage^60^. For this reason, we have analyzed contigs containing the highest and lowest %GC distribution among the entire assembly (Fig. 6).

For Y strain genome, a total of 316 contigs with %GC ≤35 were found, and 71 of those had at least one CDS (about 22%). However, in the case of Bug2148 23 contigs meeting the mentioned criterion and 17 contains a known predicted function (about 74%). Furthermore, 219 contigs containing %GC ≥65 from which 119 have functional annotation were determined for Y, and finally just 2 sequences for Bug2148 (both with annotations corresponding to hypothetical proteins).

In both cases, CDS located in GC-low regions correspond to constitutive functions such as metabolism and gene expression. Interestingly, of those genes at GC-rich locations around half of them correspond to amastigote surface proteins, a highly polymorphic and diverse family.

### Predicted single copy genes in Trypanosoma cruzi

Predicted single copy genes (SCG) encoding known protein functions from the two new genomes, were associated to specific biological processes. We found a total of 400 and 183 predicted SCG genes related to specific biological processes for Bug2148 and Y strain, respectively (Fig. S5). This figure is much higher than in previously assembled *T*. *cruzi* genomes and may have an important impact in the genetic manipulation of this parasite. These differences may be related to assembly performance and/or inherent technology capabilities as we have described before, but due to the potential importance their existence should drive further confirmations. The identification of genetic profiles for each strain, and specifically SCG sequences may define genes that could help to understand differential behaviors trough the parasite life cycle and/or infection cycle. SCG are also more suitable for new genetic manipulation techniques such as CRISPR/Cas9 than multi-copy families constituted by multi-copy sequences. Therefore, they may constitute excellent candidates as drug targets due to their importance in many metabolic pathways.

In addition, the genome sequence from Bug2148 likely allows better resolution and conservation than Y strain, in consequence, the identification of some SCG specific to Y strain not present in Bug2148 would bring interesting outcomes regarding parasite life cycle, virulence or disease evolution of this highly virulent strain.

In this regard, we have identified 2 SCG specific to Y strain: Asparagine-linked glycosylation protein 12 (ALG12) belonging to the carbohydrate derivative biosynthetic process and the molybdenum cofactor biosynthesis protein (Moco) linked to the prosthetic group metabolic process. ALG12 is a prevalent eukaryotic enzyme involved in the co-transcriptional transfer of a pre-assembled tetradecasaccharide from a dolichyl-pyrophosphate donor to the asparagine side chain of maturing proteins in the endoplasmic reticulum (ER)^61^. On the other hand, Moco is a molybdopterin cofactor of xanthine oxidase, DMSO reductase, sulfite oxidase, nitrate reductase, and other oxidases involved in purine metabolism^62^. Notably, xanthine oxidase inhibitors as allopurinol showed trypanostatic effect in infected mice^63^.

## Conclusions

The Bug2148 genome, based on the *T. cruzi* expected haploid genome size, is the most complete to date, one of the less fragmented assembled by *de novo* reported and the only one belonging to TcV. Thus, it represents a positive contribution for future genomic and transcriptomic (such as RNAseq) analysis.

To date, no thorough comparison of the differences among annotated *T. cruzi* genomes had been performed; important genomic differences between strains and/or DTUs were found in major protein super-families. This reveals a new genomic expansion and complexity and suggests the existence of a species-specific common core genome. The top 15 more expanded families are highly variable among strains. Since these families play also multiple roles in virulence, evading vector’s and host’s defensive mechanisms, the relationship between diversity and expression with strain biological features opens a new dimension in the studies of *T. cruzi* biology. Those differences may contribute to the understanding of this kinetoplastid, and by extension of one of the most neglected tropical diseases.

We assembled the largest contig (1.3 Mb) that may be considered as the first chromosome assembled (chromosome-like, without scaffolding). As mentioned, genomes (diploid and polyploid) present complex repeat structures including tandem repeats, inverted repeats, imperfect repeats and repeats inserted within repeats^64^ that are important but represent a problem for assembly algorithms, giving in most projects very fragmented assemblies of genomes, and therefore a significant loss of information^24,65,66^. In the case of *T. cruzi*, the high frequency of repeats has caused the underrepresentation of the most important gene families implicated in crucial processes, mainly related to vector, host and disease development.

We have provided evidence of gene families and/or structural sequences collapsing assemblies at repetitive telomeric regions of the 1.3 Mb chromosome-like that had not been demonstrated before for *T. cruzi*. These families might suffer more evolutive pressure in those chromosomal locations than others.

Y and Bug2148 new genomes allowed describing many more single copy genes that previously reported (from less than a dozen to several hundreds). For the first time, a closer insight into genetic composition is investigated. Therefore, we believe that our new two *T. cruzi* genomes may positively contribute to move forward in the field.

## Methods

### Parasite cultures and DNA isolation

Vero cells were grown in RPMI medium supplemented with 5% fetal bovine serum (FBS), 100 UI/mL of antibiotics mixture, 10 µg/mL streptomycin and 2 mM glutamine at 37 °C in an atmosphere of 5% CO_2_ until the cells reached 80% confluence (after 4 days). The cell monolayer was subsequently infected with metacyclic trypomastigotes (Bug2148 cl1 and Y, 10 parasites per cell). After 4 days, the supernatant medium was collected, Vero cells and amastigotes were removed by centrifugation at 1000 g by 5 minutes. Trypomastigotes were collected by centrifugation at 1600 g for 10 minutes.

Due to the DNA sample requirements for different sequencing technologies (in length mainly), genomic DNA from Y strain trypomastigotes was isolated using the “High Pure PCR Template Preparation Kit” (Roche) and DNA from Bug2148 was isolated using Phenol-Chloroform method. Both samples were treated with DNAse-free RNAse I (ROCHE) and quantified by absorbance at 260 nm using the Nanodrop ND-1000 (Thermo Scientific). All samples showed an A260/A280 ratio higher than 2.0. In both cases kDNA mitochondrial (shorter than 20 kb) was discarded by agarose gel electrophoresis.

### Genome sequencing and data processing

Genome from Bug2148 was sequenced with Pacific Biosciences (PacBio) technologies at the Norwegian Sequencing Centre (www.sequencing.uio.no), a national technology platform hosted by the University of Oslo and supported by the “Functional Genomics” and “Infrastructure” programs of the Research Council of Norway and the Southeastern Regional Health Authorities^67^. Y strain was sequenced with Illumina MiSeq series by the Genomics facility at the Parque Científico de Madrid (PCM, Madrid, Spain). Integrity from two samples was analyzed in Bioanalizer (Agilent 2100) to confirm DNA fragmentation level larger than 20 Kb for PacBio and 900 bp for Illumina sequencing.

Genome size for DTU’s II and V has been estimated to be around 115 and 106 Mb, respectively, where approximately 20% corresponds to kDNA mitochondrial^8,37^. Based on this approach 100x of read depth coverage (RDC) were sequenced for both strains.

No overlapping Paired-end reads of 2 × 300 format and 8–15 kb of read length were obtained from Illumina and PacBio, respectively. Raw reads were subject to quality-filtering using standard processes and analyzed using FASTQC tool^68^. Illumina reads shorter than 50 bp, mean quality lower than 25 (Phred Score based) were removed, and reads longer than 250 bp were trimmed.

Reads shorter than 500 bases, quality lower than 0.8 and polymerase reads shorter than 100 bases were removed from PacBio.

### Genome assembly, assembly stats and gene prediction

Trimmed reads from Y strain were assembled using SPADES (v3.9.0)^69^, with substrings within a sequence of (k) length (K-mers) for assembly of 21, 33, 55, 77, 99 and 127 K-mers. Bug2146 was assembled using HGAP v3 (Pacific Biosciences, SMRT Analysis Software v2.3.0)^70^, seed sequence length and minimal coverage values were set to 6 kb and 15X, respectively.

Statistics from the assemblies were obtained and analyzed by linux command line program Biopieces tool kit^71^.

The percentage of GC (%GC) was calculated in each entire contig and in a windowed mode (window = 1000 pb, step = 500 pb). The %GC distribution was obtained with public custom perl scripts^72^.

Gene prediction and annotation was performed using Prodigal algorithm (v2.6.3)^73^ setting to predict just complete open reading frames (ORFs) by using standard translation eukaryotic table. Gene families were predicted by Markov cluster algorithm (MCL)^74^, functional prediction was performed by Best reciprocal BLAST (Basic Local Alignment Search Tool) hit to all proteins available on Tritryp database for *T. cruzi* strains (e-value ≤1e-5). Annotations were inspected manually when possible using IGV browser^75^. Single copy genes (SCG) and Monoglyceride lipase were identified and extracted from MCL and Blastp results.

### Sialidase genes, functional distribution and telomeric repeats among assembled contigs

Transialidase gene copy quantification, identification of functional subclasses and annotations for complex contigs (0.35 ≤ %G + C ≥0.65) were extracted from MCL and Blastp results, meanwhile telomeric regions were defined by Biopieces (patscan_seq, mismatches not allowed) and IGV Browser (find motif).

## Electronic supplementary material

Supplementary Figures

Supplementary File S1

Supplementary File S2

Supplementary File S3

## Data Availability

Genomes for Bug2148 and Y strain are available from the Genbank database accession numbers NMZN00000000 and NMZO00000000, respectively.
