# Supplementary material for: Genomic assemblies of newly sequenced Trypanosoma cruzi strains reveal new genomic expansion and greater complexity
Source: Sci Rep. 2018 Oct 2;8:14631. doi: 10.1038/s41598-018-32877-2 (PMC6168536; doi:10.1038/s41598-018-32877-2)
Supplement: Supplementary file 1 — Supplementary Figures [file 41598_2018_32877_MOESM1_ESM.pdf]

Genomic assemblies of newly sequenced *Trypanosoma cruzi* strains reveal new genomic expansion and greater complexity.

Francisco Callejas-Hernández[1], Alberto Rastrojo[1], Cristina Poveda[1], Núria Gironès*[1,2] and Manuel Fresno*[1,2]

[1] Centro de Biología Molecular Severo Ochoa, Consejo Superior de Investigaciones Científicas, Universidad Autónoma de Madrid, Cantoblanco, Madrid, Spain.

[2] Instituto Sanitario de Investigación Princesa, Madrid, Spain.

* Address correspondence to: mfresno@cbm.csic.es or ngirones@cbm.csic.es Tel: +34 91 1964565; Fax: +34 91 1964420;

Supporting Information: Figures

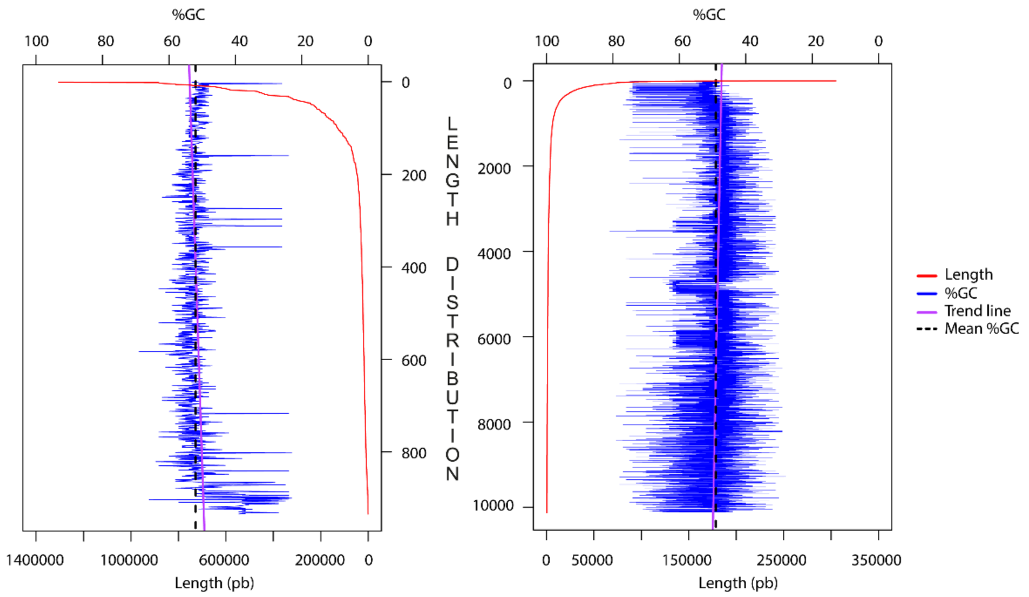

S1 Fig. %GC distribution on the contigs of the new assembled genomes. Bug2148 contigs (left panel). Y contigs (right panel). Contig length distribution (red trendline) %GC distribution (blue line) and %GC trend line (pink line).

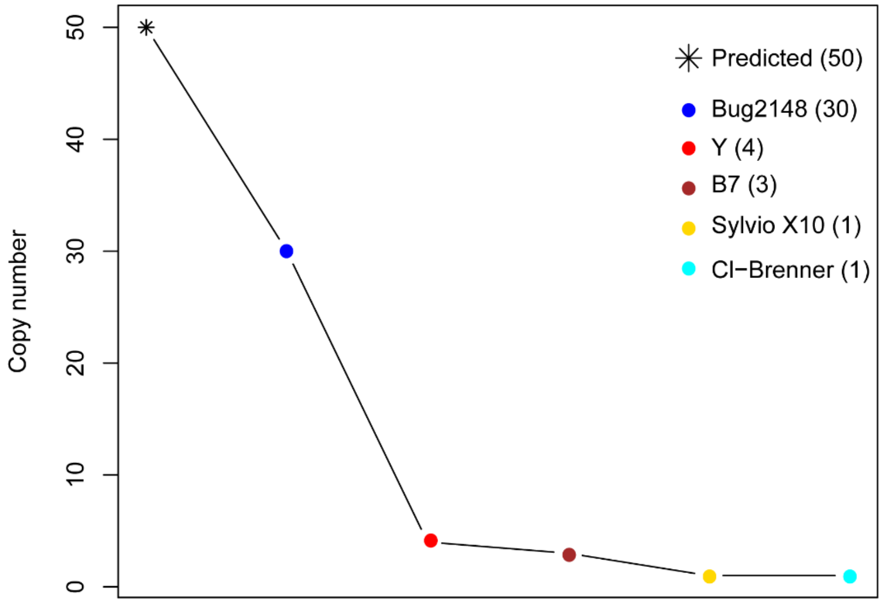

S2 Fig. MisSassembly control (Monoglyceride lipase). Copy number of Monoglyceride lipase in our new assembled and previously available genomes.

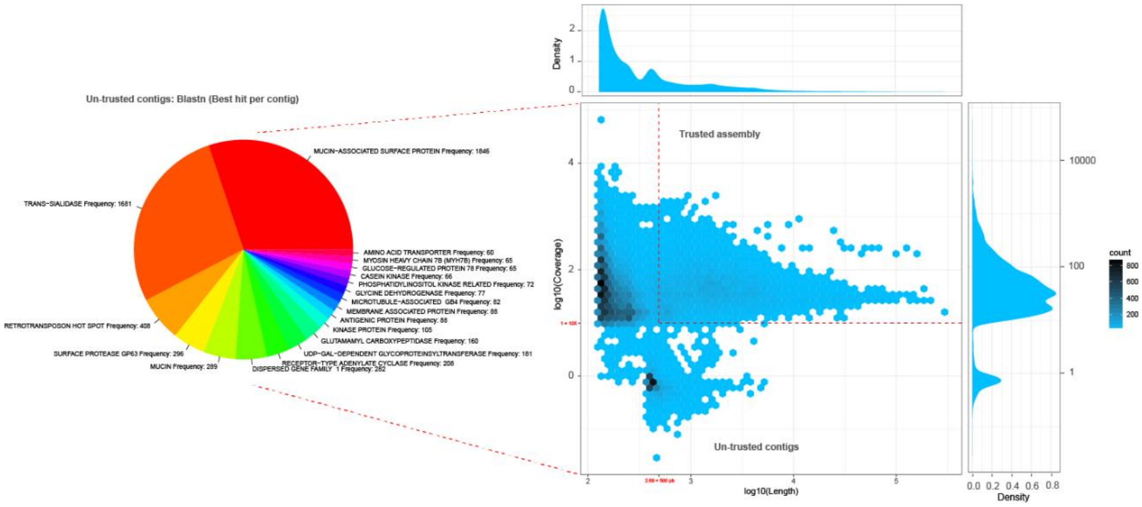

S3 Fig. Profile of contigs assembled by Illumina reads and spades assembler (Y strain), length versus coverage (log10 based). Untrusted contigs were defined as contigs with coverage below of 10X and length shorter than 500 nucleotides and therefore**,** trusted contigs constitute the assembled genome (Y strain). In this case, coverage above 10,000X is indicative of high levels of genomic plasticity and aneuploidies, in agreement to previous speculations but also it allow us to identify the specific genetic families causing these collapses.

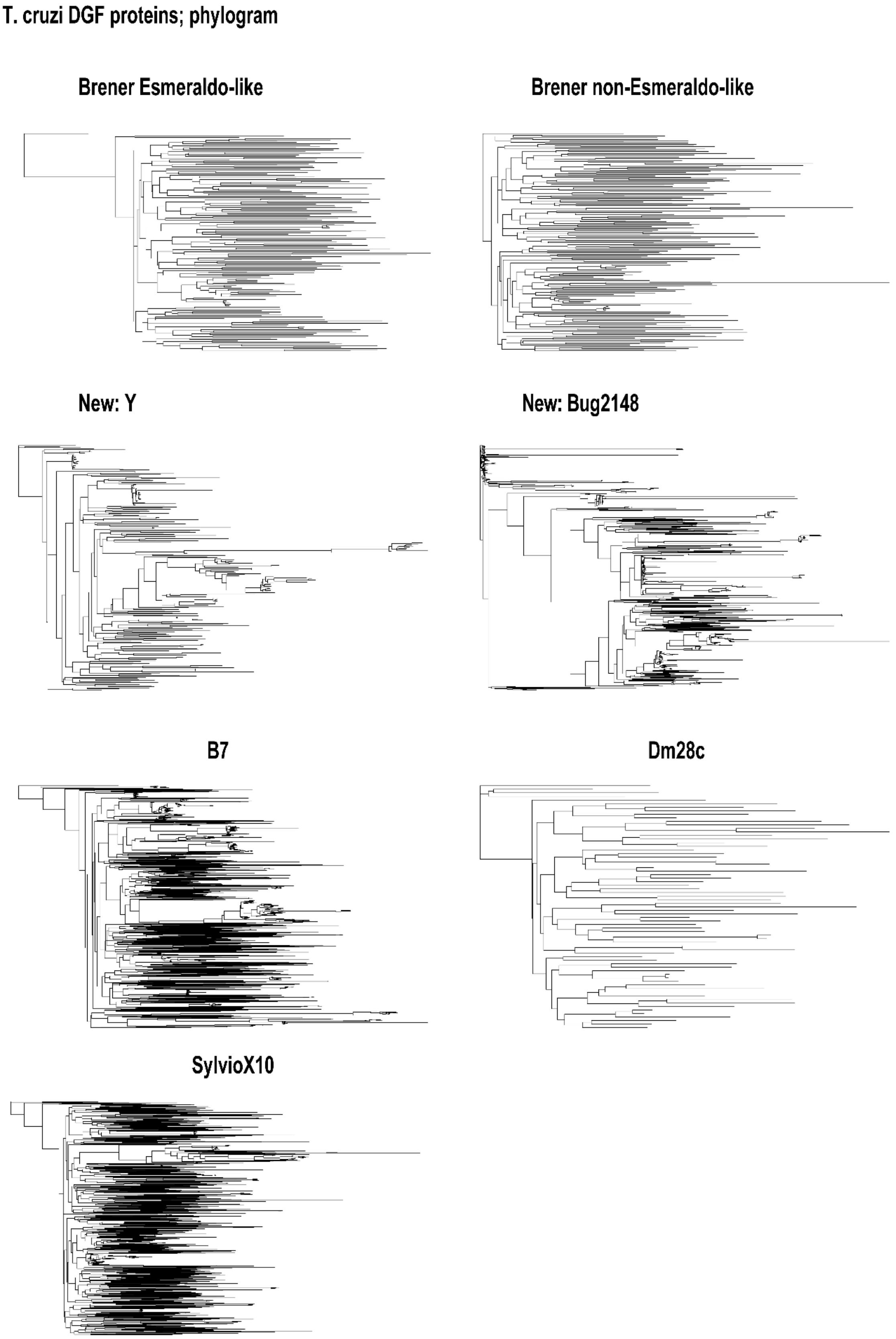

S4 Fig. Hypothetical and annotated DGF proteins in T. cruzi genomes.

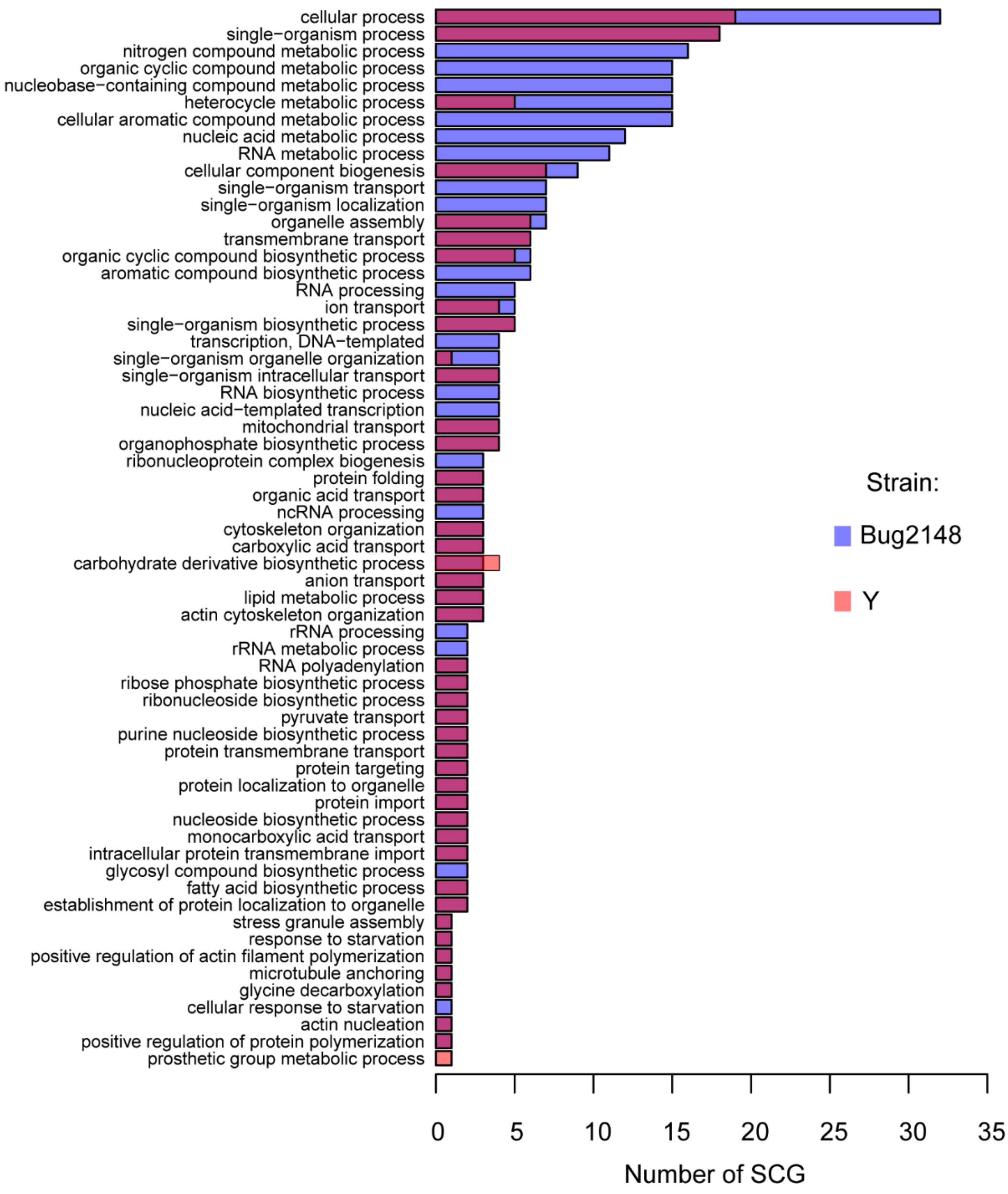

S5 Fig. SCG associated to specific biological processes. SCG association to Biological process was obtained by Gene ontology (GO) analysis in Tritryp DB (http://tritrypdb.org/tritrypdb/), redundancy was manually filtered when possible.

Supporting Information: Files

S1 File. G+C contig mean per assembled contig.

S2 File. Annotated functions in *Trypanosoma cruzi* available draft genomes.

S3 File. Contigs containing the lowest and highest linguistic complexity.
